# Irradiated Human Fibroblasts as a Substitute Feeder Layer to Irradiated Mouse 3T3 for the Culture of Human Corneal Epithelial Cells: Impact on the Stability of the Transcription Factors Sp1 and NFI

**DOI:** 10.3390/ijms20246296

**Published:** 2019-12-13

**Authors:** Gaëtan Le-Bel, Sergio Cortez Ghio, Louis-Philippe Guérin, Francis Bisson, Lucie Germain, Sylvain L. Guérin

**Affiliations:** 1 Centre LOEX de l’Université Laval, Génie Tissulaire et Régénération, Centre de Recherche du CHU de Québec -Université Laval, Axe Médecine Régénératrice, Québec, QC G1V 0A6, Canada; gaetan.lebel17@gmail.com (G.L.-B.); sergio.cortez-ghio@crchudequebec.ulaval.ca (S.C.G.); louis-philippe.guerin.1@ulaval.ca (L.-P.G.); francis.bisson.1@ulaval.ca (F.B.); Lucie.germain@fmed.ulaval.ca (L.G.); 2 Centre Universitaire d’Ophtalmologie (CUO)-Recherche, Centre de recherche FRQS du CHU de Québec, Axe Médecine Régénératrice, Québec, QC G1J 1Z4, Canada; 3 Département de Chirurgie, Faculté de médecine, Université Laval, Québec, QC G1V 0A6, Canada; 4 Département d’Ophtalmologie, Faculté de médecine, Université Laval, Québec, QC G1V 0A6, Canada

**Keywords:** human corneal epithelial cells, feeder layer, Sp1, NFI, gene profiling

## Abstract

Because of the worldwide shortage of graftable corneas, alternatives to restore visual impairments, such as the production of a functional human cornea by tissue engineering, have emerged. Self-renewal of the corneal epithelium through the maintenance of a sub-population of corneal stem cells is required to maintain the functionality of such a reconstructed cornea. We previously reported an association between stem cell differentiation and the level to which they express the transcription factors Sp1 and NFI. In this study, we investigated the impact of replacing irradiated 3T3 (i3T3) murine fibroblast feeder cells by irradiated human corneal fibroblasts (iHFL) on the expression of Sp1 and NFI and evaluated their contribution to the proliferative properties of human corneal epithelial cells (hCECs) in both monolayer cultures and human tissue engineered corneas (hTECs). hCECs co-cultured with iHFL could be maintained for up to two more passages than when they were grown with i3T3. Western Blot and electrophoretic mobility shift assays (EMSAs) revealed no significant difference in the feeder-layer dependent increase in Sp1 at both the protein and DNA binding level, respectively, between HCECs grown with either i3T3 or iHFL. On the other hand, a significant increase in the expression and DNA binding of NFI was observed at each subsequent passage when hCECs were co-cultured along with i3T3. These changes were found to result from an increased expression of the NFIA and NFIB isoforms in hCECs grown with i3T3. Exposure of hCECs to cycloheximide revealed an increased stability of NFIB that likely resulted from post-translational glycosylation of this protein when these cells were co-cultured with i3T3. In addition, iHFL were as efficient as i3T3 at preserving corneal, slow-cycling, epithelial stem cells in the basal epithelium of the reconstructed hTECs. Furthermore, we observed an increased expression of genes whose encoded products promote hCECs differentiation along several passages in hCECs co-cultured with either type of feeder layer. Therefore, the iHFL feeder layer appears to be the most effective at maintaining the proliferative properties of hCECs in culture most likely by preserving high levels of Sp1 and low levels of NFIB, which is known for its gene repressor and cell differentiation properties.

## 1. Introduction

Because of the worldwide shortage of graftable corneas that are to be used to restore visual impairments, alternatives, such as the production of a functional human cornea by tissue engineering, have emerged. In order to maintain the long-term culture of reconstructed corneas, immortalized cells were used at first [1,2]. However, both the fact that they were virally transformed and unable to accurately mimic the behavior of the native corneal cells restricted their use for corneal reconstruction.

Like the human native corneal epithelium, the tissue-engineered human cornea must support constant self-renewal of the epithelium as well as its differentiation and wound healing properties. In order to ensure these biological processes, the engineered epithelium must harbor and sustain a sub-population of corneal stem cells in the basal cell layer. To preserve these epithelial cell’s properties, the culture conditions must allow for a good proliferation of the human corneal epithelial cells (hCECs) by delaying their terminal differentiation while ensuring long-term regeneration of the epithelium once grafted on a patient. Feeder layers are a key element of these culture conditions. Indeed, co-culture of hCECs together with a feeder layer is therefore required for their amplification, as well as to preserve stem cells in primary cultures and derived cultures [3].

Co-culture of primary cells together with a feeder layer of irradiated murine 3T3 cells (i3T3) is a current practice in tissue engineering. However, mouse feeder layers were demonstrated years ago to secrete *N*-glycolylneuraminic acid (Neu5Gc) [4]. Humans are unable to produce Neu5Gc from its precursor Neu5Ac but they still retain their ability to take up Neu5Gc against which they have circulating antibodies. It has been suggested that such binding of antibody and complement to human embryonic stem cells (HESCs) would target them for death in vivo, through recognition by macrophages and natural killer cells [4]. As has been suggested for HESCs, xenogenic culture of corneal epithelial cells might therefore potentially compromise transplantation success of human corneas produced by tissue-engineering (hTECs). Moreover, varying degrees of neovascularization were reported in the peripheral cornea of patients with bilateral limbal stem cells deficiency (LSCD) that have been treated by autologous cultivated oral mucosal epithelial transplantation (COMET) when 3T3 feeder cells are used [5,6]. This phenomenon was suggested to have resulted from the fact that cultivated oral mucosal epithelial cells predominantly express pro-angiogenic factors when they are grown in the presence of 3T3 feeder cells [7,8,9]. Furthermore, 3T3 cells have an endogenous retrovirus containing a 3600-bp region of xenotropic murine leukemia virus-related virus (XMRV) which are associated with human prostate cancer and chronic fatigue syndrome [10]. Because of all these evidences, the need for producing engineered tissues that use human rather that murine feeder cells emerged, especially when such tissues are to be used for grafting purposes.

We recently used irradiated human dermal fibroblasts (iHFL) as a feeder layer in the monolayer culture of human skin keratinocytes and demonstrated that it was as efficient as i3T3 at maintaining the proliferative characteristics of these cells and the number of cell passages for which they could be grown in culture [11,12]. Meanwhile, we also previously reported an association between stem cell differentiation and their expression level of the transcription factors (TFs) Sp1 and NFI for both skin and corneal epithelial cells as well as for corneal endothelial cells [13]. Our goal herein is thus to characterize the impact of different feeder layers on the proliferative properties of hCECs in monolayer cultures.

## 2. Results

### 2.1. Morphology and Growth Characteristics of hCECs Grown with i3T3 or iHFL

**Figure 1:**
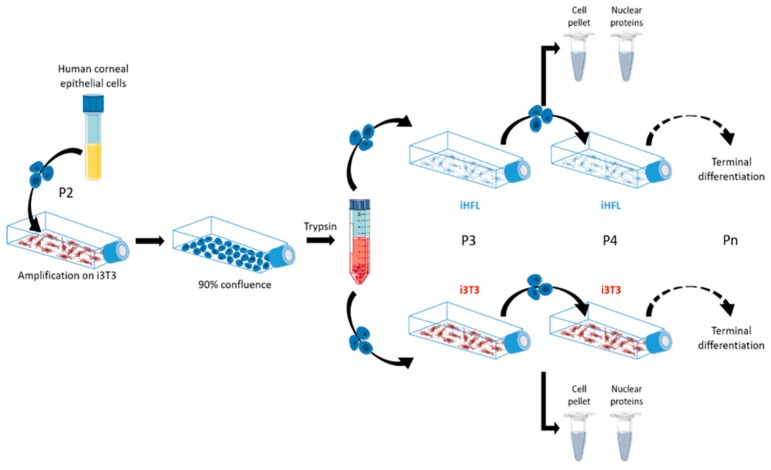
Feeder cells/hCECs co-culture conditions. Schematic representation of the procedure used for co-culturing hCECs with either iHFL or i3T3 and their use for the preparation of cell pellets and crude nuclear extracts. hCECs were also grown without any feeder cells as negative controls (not shown on Figure 1).

**Figure 2:**
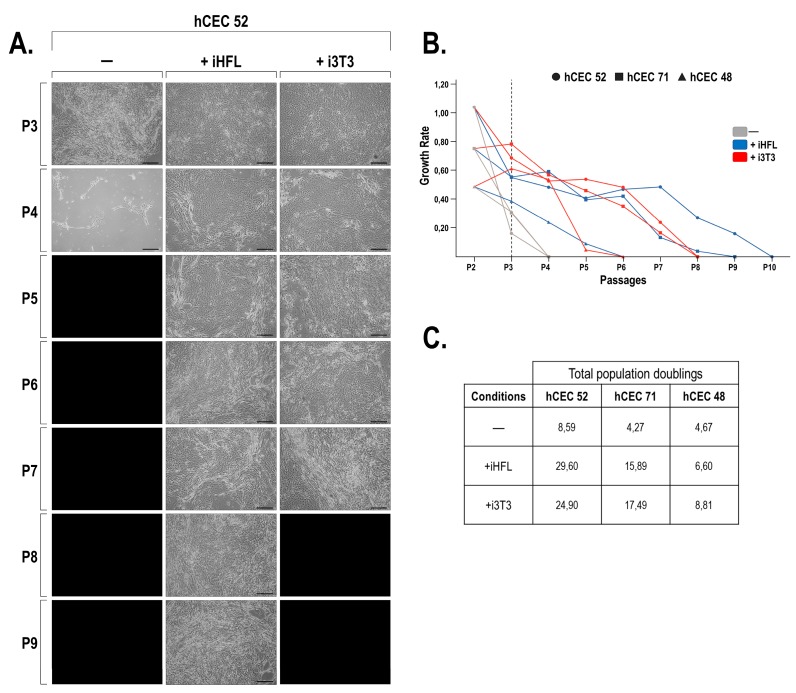
Proliferative properties of hCECs grown with iHFL or i3T3 feeder layer. (**A**) Human corneal epithelial cells (hCEC52) at passage P3 were grown either alone (-) or in the presence of feeder cells (either iHFL or i3T3) until they reached near 90% confluence before they were trypsinized and cultured to the next passage. Cells were passaged until they reach replicative senescence. Scale bars: 200 µm. (**B**) Graph representation of the growth rates calculated as the number of population doubling per day on average and determined from the hCECs cultured in panel A. (**C**) Total population doubling of hCECs grown either with (iHFL and i3T3) or without (-) a feeder layer for each of the three cell populations (hCEC48, hCEC52 and hCEC71).

In order to evaluate the impact of the feeder layer type on the morphology and growth characteristics of corneal epithelial cells, hCECs were cultured either alone or in the presence of a murine (i3T3) or human (iHFL) feeder layer (Figure 1). Three different cell populations of hCECs grown with iHFL could be cultured for up to two additional passages before they reached senescence when compared to hCECs grown with i3T3 (Figure 2 for cell population hCEC52 and Supplementary Figure S1 for cell populations hCEC48 and hCEC71). For the first two passages, cells grown with i3T3 had a slightly higher growth rate compared with hCECs grown in the presence of iHFL (Figure 2B). However, total population doublings across passages were similar between hCECs grown with either iHFL or i3T3. Overall, the more proliferative populations (hCEC52 and hCEC71) most often clustered together with both feeders, even though hCECs grown on iHFL slightly outperformed those grown on i3T3. In addition, we could not see any age-dependent impact on the growth characteristics of our three populations of hCECs (hCEC71 performed nearly as well as hCEC52, whereas hCEC48 could not be passaged beyond P6) therefore suggesting that the growth differences observed most likely relied on population inter-individual variability rather than on an age-related factor. Taken together, these results suggest that co-culturing hCECs with iHFL is as effective as culturing them with i3T3 in terms of preserving their growth and morphological characteristics in vitro. It is noteworthy that without any feeder layer, hCECs rapidly reached terminal differentiation at passage P3. Moreover, at the last passage and just prior to terminal differentiation, hCECs presented a more elongated morphology.

### 2.2. Expression of Sp1 and NFI in hCECs Grown with i3T3 or iHFL

**Figure 3:**
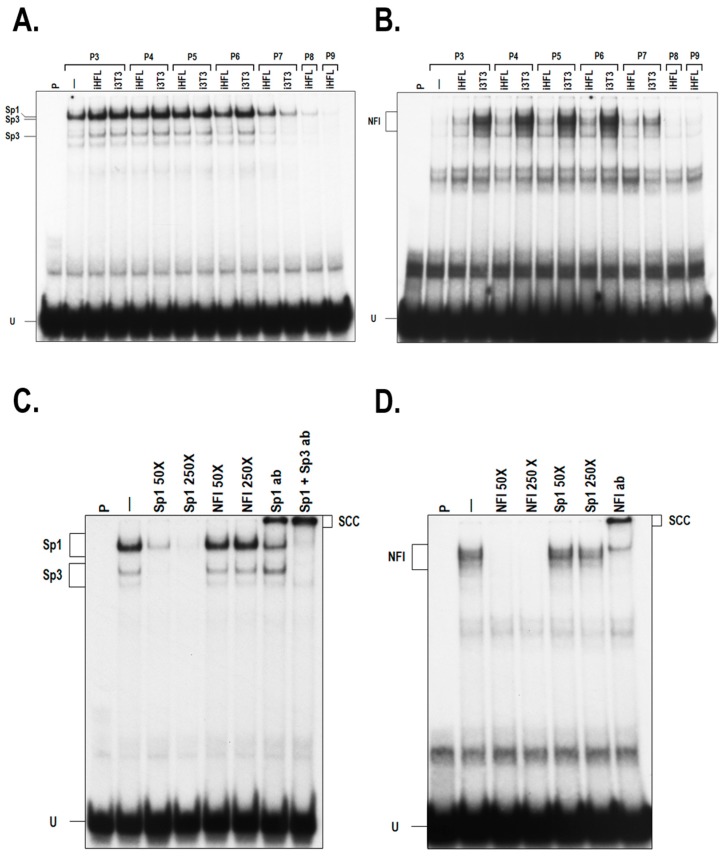
DNA binding properties of Sp1 and NFI in hCECs cultured with a feeder layer. (**A**) EMSA (Electrophoretic Mobility Shift Assay) showing the DNA binding capacity of Sp1 in hCECs (hCEC52 cell population) cultured with i3T3, iHFL, or without a feeder layer (-) over culture passages. (**B**) Nuclear extracts from panel A were used to evaluate the capacity of NFI to bind to its high affinity target site in EMSA. (**C**,**D**) Nuclear proteins from hCECs grown with iHFL at passage 3 were incubated with either the Sp1 (panel **C**) or NFI (panel **D**) labeled probe in the presence of a 50- or 250-fold molar excess of an unlabeled oligonucleotide bearing either the Sp1 or the NFI high affinity-binding site as competitor. Formation of DNA-protein complexes was then monitored by EMSA. When indicated, antibodies directed against Sp1 (Sp1 ab), Sp3 (Sp3 ab) or NFI (NFI ab) were added to the reaction mix prior to the EMSA. The position of the Sp1 and NFI complexes is indicated, as well as that of their corresponding supershifted complexes. P: labeled probe alone; -: labeled probe incubated with nuclear proteins but without unlabeled competitor; U: unbound fraction of the labeled probe.

**Figure 4:**
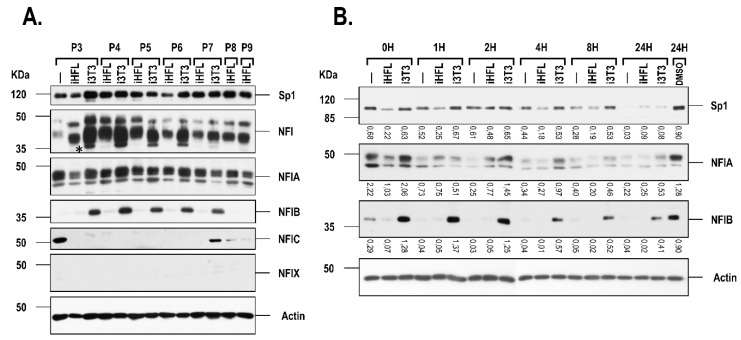
Western blot analysis of Sp1 and NFI in hCECs cultured with a feeder layer. (**A**) Nuclear extracts prepared from hCECs (hCEC52 cell population) grown in the presence of either i3T3 or iHFL at different passages (P3 to P9) were separated by SDS-PAGE and Western blotted using antibodies raised against Sp1, NFI-total (NFI), NFIA, NFIB, NFIC and NFIX. (**B**) Western blot analysis of Sp1, NFIA and NFIB in hCECs grown alone (-) or with an i3T3 or iHFL feeder layer, in the presence of either no (0H) or 50µL/mL cyclohexemide for various periods of time (1 to 24 h). As a control, cells were also incubated for 24 h with the vehicle (DMSO). The position of the appropriate molecular mass markers is indicated (kDa). Values shown beneath each blot correspond to the ratio of the TF signal over that of actin.

As we previously demonstrated a direct relationship between the endogenous expression level of the TFs Sp1 and NFI and the preservation of the growth characteristics of skin, and both corneal epithelial and endothelial cells when they are co-cultured together with i3T3 [14,15,16,17], we therefore examined whether co-culturing hCECs with iHFL as a feeder layer would also impact on the expression and DNA binding properties of these factors. Culturing hCECs with a feeder layer of either i3T3 or iHFL had little impact, if any, on the DNA binding of Sp1 (Figure 3A; also refer to Supplementary Figure S2A,B for data obtained with hCEC48 and hCEC71 cells). On the other hand, it considerably increased that of NFI (Figure 3B), as revealed by EMSA analyses. The most important increase in the NFI DNA binding activity was observed during the early passages (P3 to P6), which then progressively diminished as cells reached terminal differentiation. No significant difference was observed in the DNA binding of Sp1 between hCECs grown either with i3T3 or iHFL (Figure 3A). However, a clear difference was noted for NFI as its DNA binding capability was considerably increased in hCECs grown with i3T3 relative to such cells grown with iHFL (Figure 3B; also refer to Supplementary Figure S2C,D for data obtained with hCEC48 and hCEC71 cells). The specificity for the formation of these complexes in the EMSA was further demonstrated by the fact that formation of the Sp1 complex could only be competed by the unlabeled Sp1 oligonucleotide (Figure 3C) whereas that of the NFI complex was only prevented by the unlabeled NFI oligonucleotide (Figure 3D). Furthermore, addition of polyclonal antibodies directed against either Sp1 or NFI significantly reduced formation of these complexes in the EMSA and also yielded new, slow migrating supershifted complexes (SSC on Figure 3C,D), a clear indication that both Sp1 and NFI are present in these DNA-protein complexes in vitro. This result was further validated for Sp1 at the protein level as no significant variation of total endogenous Sp1 proteins was observed when hCECs were grown either with i3T3 or iHFL (Figure 4A, top panel; also see Supplementary Figure S3A,B). On the other hand, the use of an antibody that can recognize all NFI isoforms (NFI) (this explains the multi-bands pattern observed on Western blots) revealed a clear increase in total NFI proteins, with the appearance of an additional protein band (*) when hCECs are grown with i3T3 but not with iHFL (Figure 4A, NFI; also see Supplementary Figure S3A,B). As the NFI family comprises four different isoforms (NFIA, B, C and X) that can all recognize the same canonical DNA target sequence, we performed Western blots in order to define whether expression of any NFI isoform in hCECs is altered by the type of feeder layer used. As can be seen on Figure 4A, no significant change was noted for NFIA, which appeared as three protein bands on the blotted membrane. On the other hand, expression of NFIB was clearly higher in hCECs grown with i3T3 than in cells grown with iHFL (also see Supplementary Figure S3A,B for data obtained with hCEC48 and hCEC71 cells). Interestingly, expression of NFIC could only be detected in terminally differentiated hCECs (such as P3 hCECs grown without any feeder layer or hCECs grown with i3T3 at P7, and iHFL at P8 or P9) and not in actively proliferating cells. Consistent with the data from the gene profiling analysis (see Section 2.5 below), hCECs grown either alone or with a feeder layer (i3T3 or iHFL) did not express any NFIX protein (Figure 4A and Supplementary Figure S3A,B). We therefore conclude that the strong difference in both the DNA binding and expression of NFI observed when hCECs are grown with i3T3 can be accounted for, at least partly, by the important change in the expression of NFIB, and that expression of NFIC is closely related to terminal differentiation of these cells in vitro.

### 2.3. The i3T3 Feeder Layer Increases the Stability of NFIB

In an attempt to define the mechanisms through which NFI expression is increased in cells grown with a feeder layer in more details, we next monitored the expression of NFI (and also that of Sp1) by Western blot analysis on total proteins prepared from hCECs cultured in the presence of cycloheximide (an inhibitor of protein synthesis) for various periods of time (0 to 24h). Samples taken at different times showed a progressive decrease in the amount of Sp1 protein that reached complete suppression after a 24h exposure to cycloheximide in hCECs cultured without a feeder layer (Figure 4B; Sp1). On the other hand, the absolute amount of Sp1 remained stable until 8h of treatment in hCECs co-cultured either with i3T3 or iHFL, a low but significant level being still detectable after 24 h of treatment (Figure 4B; Sp1). Similar results were observed for NFIA, with a better over-time preservation of the NFIA protein being observed when hCECs were co-cultured with i3T3 (Figure 4B; NFI). The most striking result was observed with the NFIB protein that completely disappeared after 2h of exposure to cycloheximide in hCECs cultured without a feeder layer or with iHFL whereas nearly 33% of the NFIB protein still remained after 24h of treatment (Figure 4B; NFIB) in hCECs grown in the presence of i3T3. At the transcriptional level, no significant differences were however observed in the expression of the genes encoding Sp1 or the different NFI isoforms in hCECs grown with or without a feeder layer (Supplementary Figure S4), indicating that co-culturing hCECs with a feeder layer had no impact on the mRNA expression of these genes but rather increased their protein stability (and most particularly NFIB in hCECs co-cultured with i3T3).

### 2.4. The Presence of a Feeder Layer Alters the Glycosylation Status of Sp1 and NFI in hCECs

**Figure 5:**
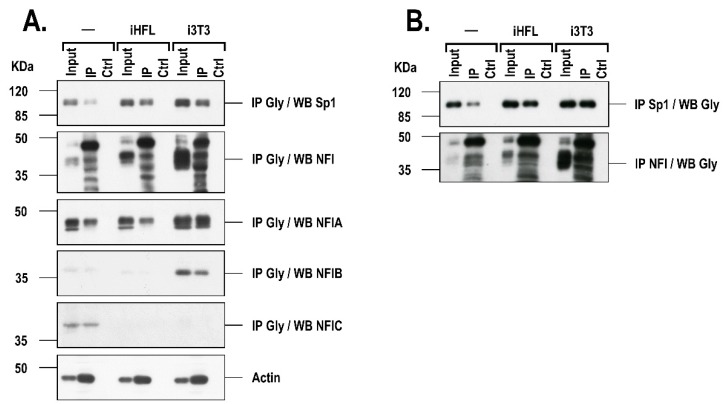
Post-translational glycosylation of Sp1 and NFI in hCECs cultured with a feeder layer. (**A**) Glycosylated proteins from nuclear extracts prepared from P3 hCECs (hCEC52 cell population) grown either alone (-) or with a feeder layer (iHFL or i3T3) were immunoprecipitated (IP) using the RL2 antibody (IP RL2) and Western blotted with the Sp1, NFI Total (NFI), NFIA, NFIB or NFIC antibodies. Actin was also blotted as a loading control. (**B**) Reverse immunoprecipitation in which both the Sp1 and NFI proteins were first immunoprecipitated and then Western blotted with the RL2 antibody. Input: nuclear extract that has not been immunoprecipitated with the RL2 antibody; Ctrl: nuclear extract immunoprecipitated with a non-specific antibody but of the same isotype as those used for the IPs.

Both Sp1 and NFI can be post-translationally altered through *O*-glycosylation [18,19,20,21,22], a process that may alter the half-life of such proteins in the nucleus. In order to verify whether the glycosylation status of Sp1 and/or NFI may differ depending on whether hCECs are co-cultured with a feeder layer, *O*-glycosylated proteins were immunoprecipitated in nuclear extracts of hCECs grown either alone or in the presence of a feeder layer (iHFL or i3T3) using an antibody (RL2) that recognizes the *O*-Linked *N*-Acetyl-d-glucosamine residues on proteins. Immunoprecipitated *O*-glycosylated proteins were then Western blotted with a polyclonal antibody that recognizes either Sp1 (IP Gly/WB Sp1) or all NFI isoforms (IP Gly/WB NFI), or with antibodies against the NFIA (polyclonal; IP Gly/WB NFIA) and both the NFIB and NFIC isoforms (monoclonals; IP Gly/WB NFIB and IP Gly/WB NFIC, respectively). Western blot analysis of the Sp1 and NFI content prior to immunoprecipitation revealed the presence of a single band with an appropriate molecular mass (95–105kDa) for Sp1, and three typical distinct protein bands (36 to 50kDa) for NFI on the gel (Figure 5A). The Sp1 protein band could easily be detected upon immunoprecipitation with the RL2 antibody in the extract from hCECs grown without a feeder layer. However, the amount of immunoprecipitated Sp1 considerably increased in the extracts from hCECs grown with iHFL or i3T3 (IP; Figure 5A). An identical pattern of immunoprecipitated NFI proteins, essentially made up of one, slow migrating predominating band together with four minor bands, was observed in hCECs cultured alone or with a feeder layer. In addition, the band intensity of some of these NFI immunoprecipitated proteins was also increased in hCECs co-cultured with i3T3. This is most likely accounted for by an increased glycosylation of both NFIA and NFIB as clearly more of these protein isoforms were immunoprecipitated in i3T3-co-cultured hCECs than in hCECs grown either alone or with iHFL (Figure 5A). Interestingly, NFIC could also be very efficiently immunoprecipitated with the RL2 antibody in P3 hCECs grown without a feeder layer but not in P3 hCECs grown with i3T3 or iHFL. The disappearance of the NFIC protein band in the input controls of HCECs grown with a feeder layer is a clear indication that the presence of either i3T3 or iHFL causes the extinction of NFIC expression in these cells. These results were further confirmed by reverse IPs in which both Sp1 and NFI were first immunoprecipitated using their specific Sp1 and NFI Abs, and then blotted with the RL2 antibody (IP Sp1/WB Gly and IP NFI/WB Gly) (Figure 5B). We therefore conclude that the nuclear ratio of glycosylated Sp1 increases when HCECs are co-cultured with either the i3T3 or iHFL feeder layer whereas glycosylated NFI only increases in HCECs grown with i3T3.

### 2.5. Gene Profiling on Microarrays

**Figure 6:**
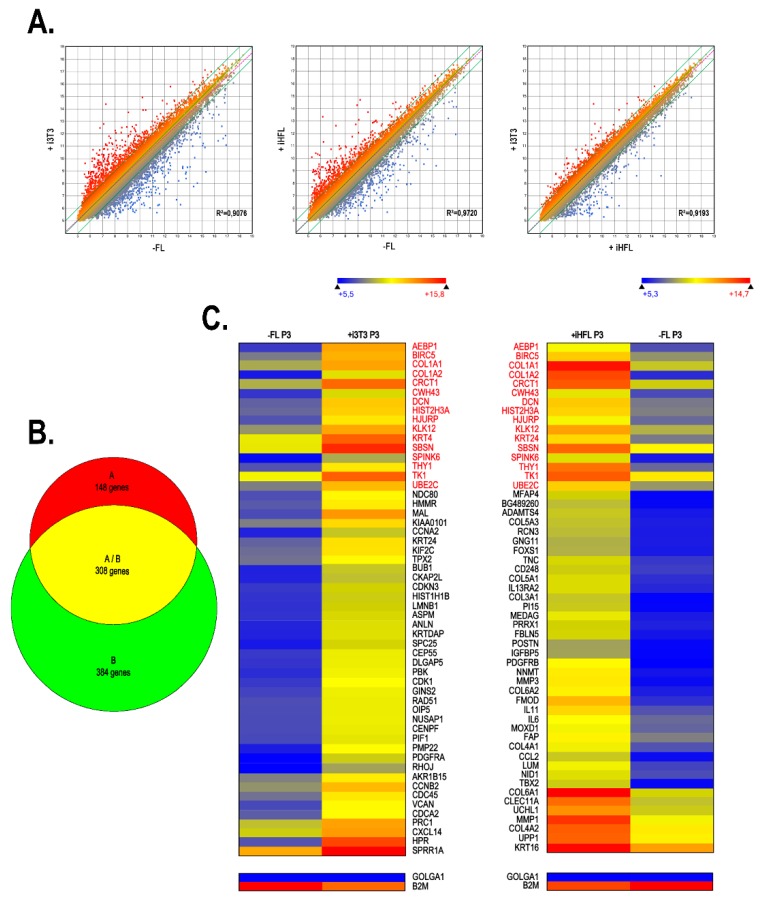
Alteration of gene expression patterns in response to the type of feeder layer used. (**A**) Scatter plots of log_2_ of signal intensity from 60,000 different targets covering the entire human transcriptome of hCECs cultured without a feeder layer (-FL; *x-axis*) plotted against hCECs grown in the presence of either i3T3 (left), or iHFL (center) (+i3T3 or +1HFL; *x-axis*). (**B**) Venn diagram that depicts the number of genes differentially regulated by more than 3-fold in hCECs cultured without a feeder layer relative to hCECs grown with i3T3 (green) or iHFL (red). Genes that are commonly regulated between these two conditions are also indicated (in yellow). (**C**) Heatmap representation of the 55 most differentially regulated genes expressed by hCECs grown with a feeder layer (i3T3 or iHFL) relative to their levels in hCECs grown with no feeder layer.

We next conducted a microarray analysis in order to compare the gene expression profiles of hCECs grown with either iHFL or i3T3. The scatter plot analysis of the 60,000 probes loaded on the chip showed moderate changes in the gene expression profiles between hCECs grown without a feeder layer and those grown with either i3T3 (Figure 6A, left panel; R^2^ = 0.9464) or iHFL (Figure 6A, right panel; R^2^ = 0.9637). We clustered all of the genes exhibiting a 3-fold or more expression variation unique to hCECs grown with a feeder layer relative to hCECs grown without a feeder layer into a Venn diagram. We identified 456 and 692 genes whose expression was differentially regulated in hCECs grown with iHFL and i3T3, respectively, relative to hCECs grown alone. Of all these genes, 308 were differentially regulated by both types of feeder layer (Figure 6B), which correspond to 67% and 44% of all the hCECs differentially regulated genes when they were grown with iHFL and i3T3, respectively. This result indicates that both feeder layers (iHFL and i3T3) tend to influence the hCECs gene expression profile in a comparable way.

We next used the Arraystar program to identify, among the microarray data files, the 55 most differentially regulated genes between the hCECs/iHFL and hCECs/i3T3 conditions (Figure 6C). However, in order to account for inter-individual variations, three different cell populations of hCECs derived from the corneas of three distinct donors were cultured either without (-FL) or with (+iHFL and +i3T3) a feeder layer and total RNA was extracted from each of them and used for microarray. Figure 6C shows gene expression means calculated from all three populations grown on either feeder layer type or cultured without one. Among these highly differentially regulated genes, 16 (*AEBP1, BIRC5, COL1A1, COL1A2, CRCT1, CWH43, DCN, HIST2H3A, HJURP, KLK12, KRT4, SBSN, SPINK6, THY1, TK1 and UBE2C;* identified in red on Figure 6C) were similarly differentially regulated (all had their expression increased by the feeder layer) when the data from the –FL/+i3T3 and –FL/+iHFL conditions were compared with each other (gene names in red on Figure 6C).

### 2.6. The Feeder Layer Preserves the Population of Corneal Epithelial Stem Cells in Tissue-Engineered Human Corneas

**Figure 7:**
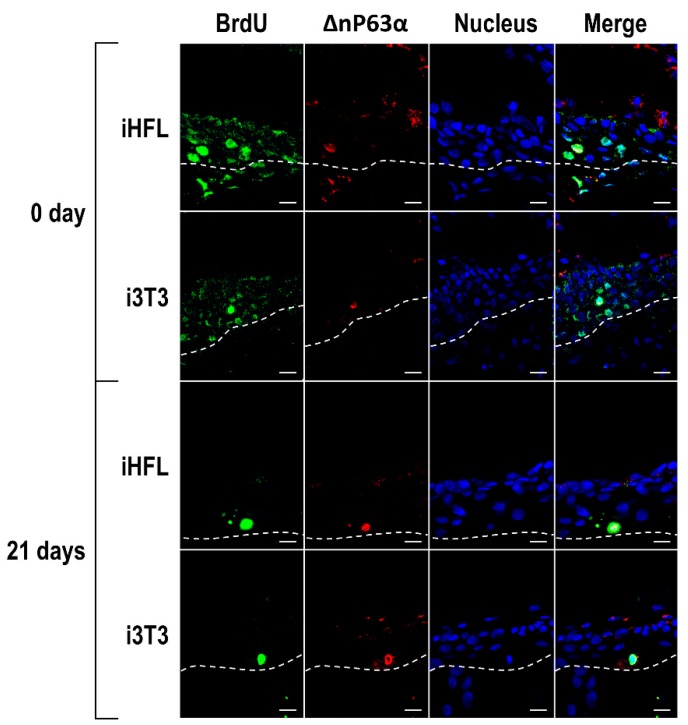
Detection of human corneal epithelial stem cells in the tissue engineered cornea. Indirect immunofluorescence analysis of BrdU (green labeling) and ΔNp63α (red labeling) to assess the presence of stem cells in the basal layer (dotted line) of the tissue-engineered epithelia produced using hCECs grown with either i3T3 or iHFL as a feeder layer at 0 and 21 days following interruption of the BrdU treatment. Nuclei were counterstained with Hoechst 33,258 reagent and appear in blue. Scale bar: 20 µm.

In order to determine whether iHFL are as efficient as i3T3 at preserving the corneal stem cells population in the stratified corneal epithelium, we cultured hCECs in the presence of either i3T3 or iHFL and then used these epithelial cells to produce human tissue-engineered corneas (hTECs) by the self-assembly approach [23]. Following maturation at the air-liquid interface for 7 days (to allow the complete stratification of the corneal epithelium), hTECs were labeled with 10 µM of the thymidine analog 5-bromo-2’-deoxyuridine (BrdU) for 7 days and chased for 0 to 21 days with BrdU free medium, a procedure that is currently used to identify slow-cycling or mitotically quiescent ‘label-retaining’ stem cells [24,25]. Once such cells have been labeled, they will retain BrdU for a much longer period of time whereas the label will be progressively lost through multiple mitoses in more differentiated transient amplifying cells that are mitotically active. BrdU-positive cells could be observed in the basal cell layer of both the hTECs produced using hCECs co-cultured either with i3T3 or iHFL at day 0 (Figure 7; top panel) and remained present at approximately the same cell density at day 21 (Figure 7; bottom panel; also see Supplementary Figure S5 for data obtained at day 7 and 14). No difference was observed in the proportion of corneal epithelial stem cells between hTECs produced using hCECs grown with i3T3 or iHFL. These BrdU-positive cells also stained positive for the intermediate filament ΔNp63α, a well known marker of corneal limbal stem cells [26] (Figure 7 and Supplementary Figure S5; merge). These results suggest that co-culturing hCECs together with iHFL is as efficient as culturing them with i3T3 at preserving corneal, slow cycling, epithelial stem cells that still stain positive for both BrdU and ΔNp63α in the basal epithelium of the reconstructed hTECs after 21 days following the BrdU treatment.

### 2.7. In Silico Prediction of Biological Functions Whose Regulation is Modified in hCECs Cultured with iHFL through Gene Interaction Network Analyses.

Using the Network Analyst platform, we next filtered, normalized, and performed a pairwise differential expression analysis on the microarray linear expression data from three different populations of hCECs cultured either on iHFL or on i3T3. We identified a total of 198 statistically differentially expressed genes between these conditions (adjusted p-value < 0.05 and logFC > 1.5). The results from this analysis were then uploaded into the Ingenuity Pathway Analysis (IPA) software to be further analyzed.

**Figure 8:**
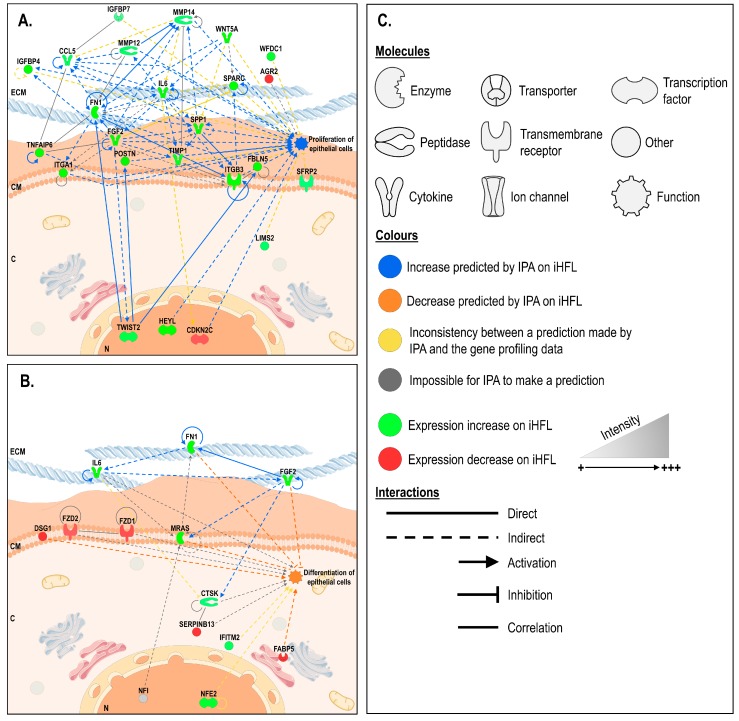
Ingenuity pathway analysis (IPA) of gene interaction networks altered by the iHFL feeder layer built around biological functions of interest: the ‘proliferation’ (panel **A**) and ‘differentiation’ (panel **B**) of hCECs. (**C**) ECM: extracellular matrix; CM: cell membrane; C; Cytoplasm; N: nucleus.

IPA’s statistical algorithms and curated knowledge database can be used to predict what and how biological functions are likely to be influenced when provided with data from a differential expression analysis. We thus selected two biological functions of interest, “proliferation” and “differentiation” of epithelial cells, to which we connected all the genes that were linked to these functions according to the database, but that were also significantly differentially expressed in our dataset (Figure 8; data for both the “adhesion” and “migration” biological functions are shown in Supplementary Figure S6). We then used IPA to examine how these genes interacted and to computationally predict how the resulting networks affected the biological functions of interest. IPA predicted that hCECs would adhere, migrate and proliferate more but differentiate less, when they are grown on iHFL rather than i3T3, given our microarray data analysis.

## 3. Discussion

Blindness resulting from corneal opacification, which accounts for nearly 4% of all cases of blindness, is estimated to affect 1.5 million people worldwide [27]. In such cases, corneal transplantation is particularly effective at helping these patients recover eyesight but the insufficient availability of cornea donors considerably restricts its curative potential. A recent global survey estimated the severe shortage in graftable corneas to 1 cornea available for every 70 needed [28,29] therefore highlighting the importance of exploring the use of alternative approaches, such as tissue reconstruction, to fulfill such needs. In that respect, co-culturing primary cells that are to be used for tissue engineering together with a feeder layer of irradiated murine 3T3 cells proved particularly effective at preserving the growth and morphological characteristics of cells such as hCECs that otherwise could not be cultured for more than a few passages in vitro [3]. However, alternatives to the use of animal cells as feeder layers should be considered when one wish to use such primary cultured cells for clinical purposes. With the long-term objective of developing a human tissue-engineered cornea (hTEC) that would be suitable for grafting, we investigated the impact of replacing i3T3 by irradiated human fibroblasts (iHFLs) on the properties of hCECs that are used in the production of hTECs. In this study we demonstrated that i3T3 can efficiently be substituted by iHFL for primary culture of hCECs. The use of iHFL as a feeder layer also improved the number of cell passages hCECs can sustain and improved the protein stability of the TFs Sp1 and NFI that also play key functions in the growth and migratory properties of these cells [16,30].

**Figure 9:**
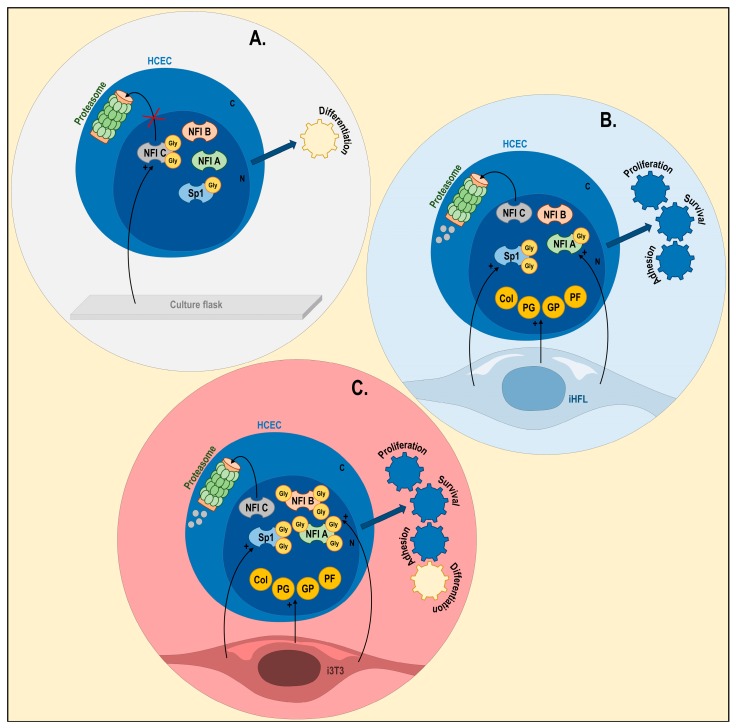
Impact of the feeder layer type on the characteristics of hCECs in vitro. hCECs grown without any feeder layer (panel **A**), with iHFL (panel **B**) or with i3T3 (panel **C**). Transcription factors Sp1, NFIA, NFIB and NFIC are depicted. Gly: glycosylation; Col: collagens; PG: proteoglycans; GP: glycoproteins; PF: proliferation factors; C: Cytoplasm; N: nucleus.

We have previously shown that the feeder layer-dependent (i3T3) preservation of the growth characteristics of primary cultured skin keratinocytes and both corneal epithelial and endothelial cells also rely on maintaining the stability of the TFs Sp1 and NFI [14,15,16,17]. Both Sp1 and NFI are well known housekeeping transcription factors that each plays a very important role in cell proliferation [31,32,33,34]. Remarkably, i3T3 were proposed to delay terminal differentiation of human skin keratinocytes by preventing the proteosomal degradation of Sp1 and NFI, both of which play pivotal functions during cell cycle progression [14,33,35]. As for i3T3, co-culturing hCECs with iHFL as a feeder layer also considerably improved the DNA binding properties of Sp1, although no significant difference could be observed between both types of feeder layer. On the other hand, culturing hCECs in the presence of i3T3 had a much greater impact than iHFL on both the expression and DNA binding of NFI, a result that can also be explained essentially by an i3T3-dependent increase in the expression of the NFIB isoform (Figure 4A; also see Figure 9). This increase in NFIB expression is puzzling in that NFIB was recently identified as a new oncogene in small cell lung cancer [36,37,38] and most particularly in triple negative breast cancer (TNBC), where it contributes to cell proliferation/survival through a direct suppression of p21 transcription [39]. NFIB has been recognized as a TF that can either promote or suppress human cancers in a cancer-type dependent manner [40,41]. This factor has also been shown to play a particularly important role as a coordinator of both epithelial and melanocyte stem cell behavior in the skin, a process dependent on the regulatory action of endothelin-2 (Edn2) and whose expression is negatively regulated by NFIB [42].

Exposure of hCECs to cyclohexemide provided evidence that the increased amount of NFIB observed in hCECs grown with i3T3 do correlate with an increased half-life of this protein rather than with an increase in its gene transcription. Furthermore, this NFIB protein with an extended half-life was also shown to be hyper-glycosylated, a post-translational modification that may contribute at increasing the stability of NFIB by preventing its proteosomal degradation as we recently reported for skin keratinocytes [14] (summarized on Figure 9). Glycosylation of NFI proteins has been reported previously for both NFIB and NFIC. Indeed, NFIC has been shown to be *N*-glycosylated in the mouse mammary gland [21] whereas glycosylation of the C-terminus domain of the NFIB2 isoform was demonstrated to alter its cooperation with other TFs expressed by JEG-3 cells, such as STAT5 [22]. Interestingly, expression of NFIC could only be observed in hCECs that had reached near terminal differentiation. Loss of NFIC expression in knockout mice has also been reported to prevent proper differentiation of odontoblasts, leading to the formation of aberrant odontoblasts in the early stage of root formation of molar teeth [43]. Osteoblast differentiation has also been shown to be under the regulatory influence of NFI through activation of the Runt-related transcription factor 2 (Runx2)-NFIC- Specificity protein 7 (Sp7; also called Osterix (Osx)) pathway [44]. It would be interesting to determine whether NFIC somehow contributes to the progression of the corneal basal epithelial cells into the more differentiated, less mitotically active suprabasal epithelial cells.

The use of iHFL as a feeder layer proved to be as effective at maintaining the proliferative capacity of hCECs as when they are co-cultured with i3T3. Interestingly, replacing i3T3 by iHFL as a feeder layer had a much reduced impact on the hCECs transcriptome. Indeed, we found that only 456 genes are differentially regulated by more than a 3-fold factor when hCECs are grown on iHFL relative to hCECs grown without a feeder layer, compared to 692 when such cells are co-cultured on i3T3. It is also particularly interesting to note that more than 67% of the hCECs/iHFL differentially regulated genes (total of 308) were also found as similarly deregulated in hCECs/i3T3. In addition, 16 (29%) out of the 55 most differentially regulated genes appearing on the heatmap from Figure 6C are also shared by hCECs/iHFL and hCECs/i3T3. It is noteworthy that among the 55 most differentially regulated genes from the –FL/+iHFL condition, 19 genes encode proteins related either to the extracellular matrix (ECM) organization (9 collagen genes (*COL1A1, COL1A2, COL3A1, COL4A1, COL4A2, COL5A1, COL5A3, COL6A1* and *COL6A2*), 3 proteoglycan genes (decorin (*DCN*), fibromodulin (*FMOD*), lumican (*LUM*)), 4 glycoproteins (nidogen-1 (*NID-1*), tenascin C (*TNC*), fibulin-5 (*FBLN5*), periostin (*POSTN*)), or to ECM remodeling (matrix metalloproteinases *MMP1* and *MMP3*, and *a disintegrin and metalloproteinase with thrombospondin motifs 4* (*ADAMTS4*)) (Figure 9).

The marked increase in thymidine kinase 1 (TK1) gene expression in hCECs grown with a feeder layer, whose protein product is predominantly synthesized in the cell during the S phase of the cell cycle, is consistent with the need to increase DNA synthesis in response to the growth response induced by the feeder layer. This is also in agreement with the particularly elevated expression of the ubiquitin-conjugating enzyme E2C (UBE2C) in hCECs grown with a feeder layer as this enzyme, an important component of the proteasome system, is required for the degradation of mitotic cyclins and the maintenance of the cell cycle progression [45,46]. Furthermore, and consistent with the increased proliferative capacity of hCECs that are grown with a feeder layer, knockdown of the genes encoding the transcriptional repressor adipocyte enhancer binding protein 1 (AEBP1) and survivin (BIRC5), and whose expression is also markedly increased in hCECs grown with iHFL or i3T3 (Figure 6C), was recently shown to considerably suppress the proliferation, migration and epithelial-mesenchymal transition of gastric and ovarian cancer cells, respectively [47,48]. The HJURP (Holliday junction recognition protein) gene, whose transcription is also strongly increased by the presence of a feeder layer, encodes a centromeric histone chaperone that helps recruit the CenH3 (CENP-A) histone H3 variant. Knockdown experiments provided evidence that HJURP could regulate bladder cancer cell’s proliferation and apoptosis via the PPARγ-SIRT1 negative feedback loop [49]. Interestingly, HJURP was also proposed as a regulator of cellular senescence through a p53-dependent pathway and might thereby contribute to tissue aging and protection of cellular transformation [50].

Functional analyses of the transcriptional profiles also suggested that co-culturing hCECs with iHFL rather than with i3T3 could translate into an increase in hCECs culture lifetime. In silico directional prediction of biological processes of interest, such as proliferation and differentiation, revealed that the mean gene expression profile of hCECs cultured on iHFL would likely promote epithelial cell growth and inhibit epithelial cell differentiation when compared to hCECs cultured on i3T3. As there were discrepancies between these predictions and the results of our in vitro experiments, it is likely that other molecular mechanisms, such as cell migration and adhesion, may contribute, at least in part, to the enhanced hCEC culture lifetime observed when they are grown with an iHFL feeder layer (Figure 9). It would thus surely prove interesting in the future to explore how some of the key differentially regulated genes we identified herein could influence the characteristics of hCECs’ cultures.

## 4. Materials and Methods

This study was conducted in agreement with the Helsinki declaration and was performed under the guidelines of the research ethics committee of the ‘CHU de Québec’ (ethic code: DR-002-955, protocol renewal approved on 31 January 2018). All patients were given adequate information to provide written consents.

### 4.1. Cell Culture and Production of Tissue-Engineered Human Cornea

Human corneal epithelial cells (hCECs) were isolated from the limbal area of normal eyes (obtained from the Banque d’Yeux Nationale of the Centre Universitaire d’Ophtalmologie; CHU de Québec - Université Laval Hospital, Québec, QC, Canada) of 48-, 52- and 71 year-old donors as previously reported [30,51]. hCECs were cultured from passages 3 to 9 with a feeder layer of either irradiated murine Swiss-3T3 fibroblasts (i3T3; ATCC, Rockville, MD) [51] or irradiated human fibroblasts (iHFL) isolated from the foreskin of a 10 day-old donor and cultured in ckDME-Ham as recently described [11,16] (Figure 1). They were seeded at 5 × 10^5^ cells/T75 tissue culture flasks together with either i3T3 (seeded at 1.5 × 10^6^ cells/T75 flask) or iHFL (seeded at 4.5 × 10^5^ cells/T75 flask). Once hCECs reached 80–90 confluence, they were trypsinized and re-seeded to the next passage in T75 flasks. All cells were grown under 8% CO_2_ at 37 °C and culture medium was changed after 2–3 days [22]. Cells were photographed using a Nikon Eclipse TS100 (Nikon Canada, Mississauga, ON, Canada) equipped with a numeric CCD camera (AxioCam 105 Color; Zeiss). At each passage, cell number was counted with a Beckman Coulter. Growth rates were calculated using the following formula: Population doubling = log ((number of cells obtained)/(number of cells seeded))/log (2). The two-layers human tissue-engineered corneas (hTECs) were produced following the self-assembly approach and maintained in DH medium ((Dulbecco-Vogt modification of Eagle’s medium with Ham’s F12 in a 3 : 1 ratio) with supplements (5% FetalClone II serum, 5 μg/mL insulin, 0.4 μg/mL hydrocortisone, 10 ng/mL epidermal growth factor, 10^−10^ mol/L cholera toxin, 100 µg/mL Penicilin, and 25 µg/mL Gentamycin)) under 8% CO_2_ at 37 °C as previously described [22,52].

### 4.2. Cycloheximide Chase Assay

Cycloheximide (Sigma Aldrich, Saint Louis, MO, USA) was added to DH medium at a concentration of 50 μg/mL. hCECs were then harvested 0, 1, 2, 4, 8 and 24 h after addition of cycloheximide and processed for the preparation of crude nuclear proteins as described in Section 4.4

### 4.3. BrdU Labeling and Immunofluorescence Analyses

hTECs were incubated with fresh DH medium containing 10 μM 5-bromo-2’-deoxyuridine (BrdU; Sigma Chemicals, St-Louis, MO) for 7 days (hTECs were fed fresh medium supplemented with 10 μM BrdU every 48 h). hTECs were then chased for 0, 7, 14 and 21 days by switching to BrdU free medium. Detection of both BrdU and ΔNp63α was conducted by immunofluorescence analyses performed as described [53] on 10-µm thick cryosections fixed for 10 min. at room temperature with 1% formol and 10 min. at −20 °C with methanol and in NaOH (0.07 N) for 10 min. at room temperature. The following primary antibodies were used: anti-human ΔNp63α (clone 250, 1:2000; rabbit polyclonal produced by MÉDIMABS, Montréal, Qc, Canada (not commercially available)) conjugated to cyanine 3, and a mouse monoclonal anti-BrdU antibody (347580 (monoclonal), 1:500; BD Pharmingen). Rabbit anti-mouse IgG H+L antibodies conjugated with Alexa 488 (A11059, 1:500; Invitrogen) and goat anti-rabbit IgG H+L antibodies conjugated with Alexa 594 (A11012, 1:1000; Invitrogen) were used as secondary antibodies. Cell nuclei were counterstained with Hoechst reagent 33,258 (1:100; Sigma Chemicals). Negligible background was observed for controls (primary antibodies omitted). Fluorescence was observed using a confocal microscope (Zeiss Imager. Z2 LSM 800; Zeiss Canada Ltd., North York, ON, Canada).

### 4.4. Nuclear Extracts and EMSA (Electrophoretic Mobility-Shift Assay)

hCECs at passages 3 to 9 were seeded in the presence of either iHFL or i3T3 at 7 × 10^3^ cells/cm^2^ in 75 cm^2^ tissue-culture flasks and grown for four days at 37 °C until they reached near-confluence (which corresponds to 90% coverage of the culture flask). As a negative control, hCECs at the same passages were also seeded alone. Nuclear extracts were prepared from all cultured cells, dialyzed, and kept frozen in small aliquots at −80 °C as previously described [54]. Electrophoretic mobility shift assays (EMSA) were conducted with double-stranded oligonucleotides bearing the DNA binding site for either Sp1 (top strand: 5′-GATCATATCTGCGGGGCGGGGCAGA CACAG-3′; bottom strand: 5′-GATCC TGTGTCTGCCCCGCCCCGCAGATAT-3′) or NFI (top strand: 5′- TTATTTTGGATTGAAGCCAATATGAG-3′; bottom strand: 5′- CTCATATTGGCTTCAATCCAAAATAA -3′) as previously described [55].

### 4.5. Immunoprecipitation and Western blots

Immunoprecipitation (IP) of glycosylated NFI and Sp1 proteins was performed using an antibody raised against *O*-linked GlcNAc residues (MA-072 RL2 (monoclonal), 4 µg; Invitrogen) on 500 µg nuclear proteins from hCECs (P3) grown with or without a feeder layer (i3T3 or iHFL). For negative controls, IPs were performed using either a rabbit (SC-2027 (monoclonal), 4 µg; Santa Cruz Biotechnology) or a mouse (X0931 (monoclonal), 4 µg; Dako) isotype antibody. Reverse IPs were conducted on both Sp1 and NFI as previously described [14,56]. For Western blot analyses, 10 µg nuclear proteins were added to 4X sample buffer and then size-fractionated on an 8% SDS-PAGE before being transferred onto a nitrocellulose membrane blotted as described [57]. Western blot analyses were conducted, as described [58] with the same nuclear extracts as those used for EMSA, and using primary antibodies directed against the following proteins: total NFI (sc-5567 (polyclonal), 1:2000; Santa Cruz Biotechnology), NFI-A (ab11988 (polyclonal), 1:500; Abcam, Toronto, ON, Canada), NFI-B (ab51352-100 (monoclonal), 1:100; Abcam), NFI-C (ab89516 (monoclonal), 1:500; Abcam), NFI-X (ab67169 (polyclonal), 1:100; Abcam), Sp1 (Sc-59 (polyclonal) 1:250; Santa Cruz Biotechnology), actin (1:40000; Santa Cruz Biotechnology, Dallas, TX, USA), and a peroxidase-conjugated AffiniPure Goat secondary antibody against mouse IgG or rabbit IgG (1:2500 dilution; Jackson ImmunoResearch Laboratories, West Grove, PA, USA). For IPs, a secondary antibody True Blot anti-mouse HRP properties (18-8817-31 (monoclonal), 1:1000; Rockland) and a secondary antibody True Blot anti-rabbit HRP properties (18-8816-31 (monoclonal), 1:1000; Rockland) were used. The labeling was revealed using an ECL Plus Western Blotting Detection Reagent Kit (Amersham, Pharmacia Biotech Co., Uppsala, Sweden) [59,60].

### 4.6. Gene Expression Profiling

All microarray analyses were conducted by the CUO-Recherche gene profiling service (Québec, QC, Canada), exactly as recently described [61]. As biological replicates, total RNA was obtained from 3 different populations of hCECs (hCEC-48, -52 and -71) grown either with or without i3T3 or iHFL. All data generated from the arrays were also analyzed by robust multi-array analysis (RMA) for background correction of the raw values. They were then transformed in Log2 base and quantile normalized before a linear model was fitted to the normalized data to obtain an expression measure for each probe set on each array. Scatter plots and heat maps were generated using the ArrayStar V4.1 (DNASTAR, Madison, WI, USA) software. All microarray data presented in this study comply with the *Minimum Information About a Microarray Experiment* (MIAME) requirements (GEO# GSE141841; https://www.ncbi.nlm.nih.gov/geo/query/acc.cgi?acc=GSE141841)

### 4.7. Bioinformatics and Statistical Analyses

The *ArrayStar* microarray linear expression data (hCEC-48, -52, -71 grown with i3T3 or iHFL) were uploaded into *Network Analyst* (https://www.networkanalyst.ca/), a web tool based on the R language through which they were filtered and normalized (variance stabilizing normalization). A pairwise differential gene expression analysis was then carried out using the limma statistical method, which resulted in a list of statistically differentially expressed genes (adjusted *p*-value<0.05 and LogFC>1.0) [22]. This list was then uploaded to and analyzed with *Ingenuity Pathway Analysis* (IPA; QIAGEN Inc., https://www.qiagenbioinformatics.com/products/ingenuitypathway-analysis) software in order to compute and visualize causal gene interaction networks around selected cellular functions of interest in hCECs [62].

## Supplementary Materials

The following are available online at https://www.mdpi.com/1422-0067/20/24/6296/s1.
