# Supplementary material for: Irradiated Human Fibroblasts as a Substitute Feeder Layer to Irradiated Mouse 3T3 for the Culture of Human Corneal Epithelial Cells: Impact on the Stability of the Transcription Factors Sp1 and NFI
Source: Int J Mol Sci. 2019 Dec 13;20(24):6296. doi: 10.3390/ijms20246296 (PMC6940969; doi:10.3390/ijms20246296)
Supplement: Supplementary file 1 [file ijms-20-06296-s001.pdf]

SUPPLEMENTARY MATERIAL:

Irradiated human fibroblasts as a substitute feeder layer to irradiated mouse 3T3 for the culture of human corneal epithelial cells: impact on the stability of the transcription factors Sp1 and NFI

Gaëtan Le-Bel[1,2,4], Sergio Cortez Ghio[1,4], Louis-Philippe Guérin[1,3], Francis Bisson [1,4], Lucie Germain[1,4] and Sylvain L. Guérin [1,2,3]*

[1] Centre LOEX de l'Université Laval, Génie Tissulaire et Régénération, Centre de Recherche FRQS du CHU de Québec, Axe Médecine Régénératrice, Québec, QC, Canada; genevieve.rioux.9@ulaval.ca (G.R.)

[2] Centre Universitaire d'Ophtalmologie (CUO)-Recherche, Centre de recherche FRQS du CHU de Québec, Axe Médecine Régénératrice, Québec, QC, Canada; manal.benhassine.1@ulaval.ca (M.B.); sylvain.guerin@fmed.ulaval.ca (S.L.G)

[3] Département d'Ophtalmologie, Université Laval, Québec, QC, Canada;

[4] Département de Chirurgie, Université Laval, Québec, QC, Canada;

* Corresponding author; Tel.: +1-418-682-7565; E-mail: Sylvain.guerin@fmed.ulaval.ca

Supplementary figures

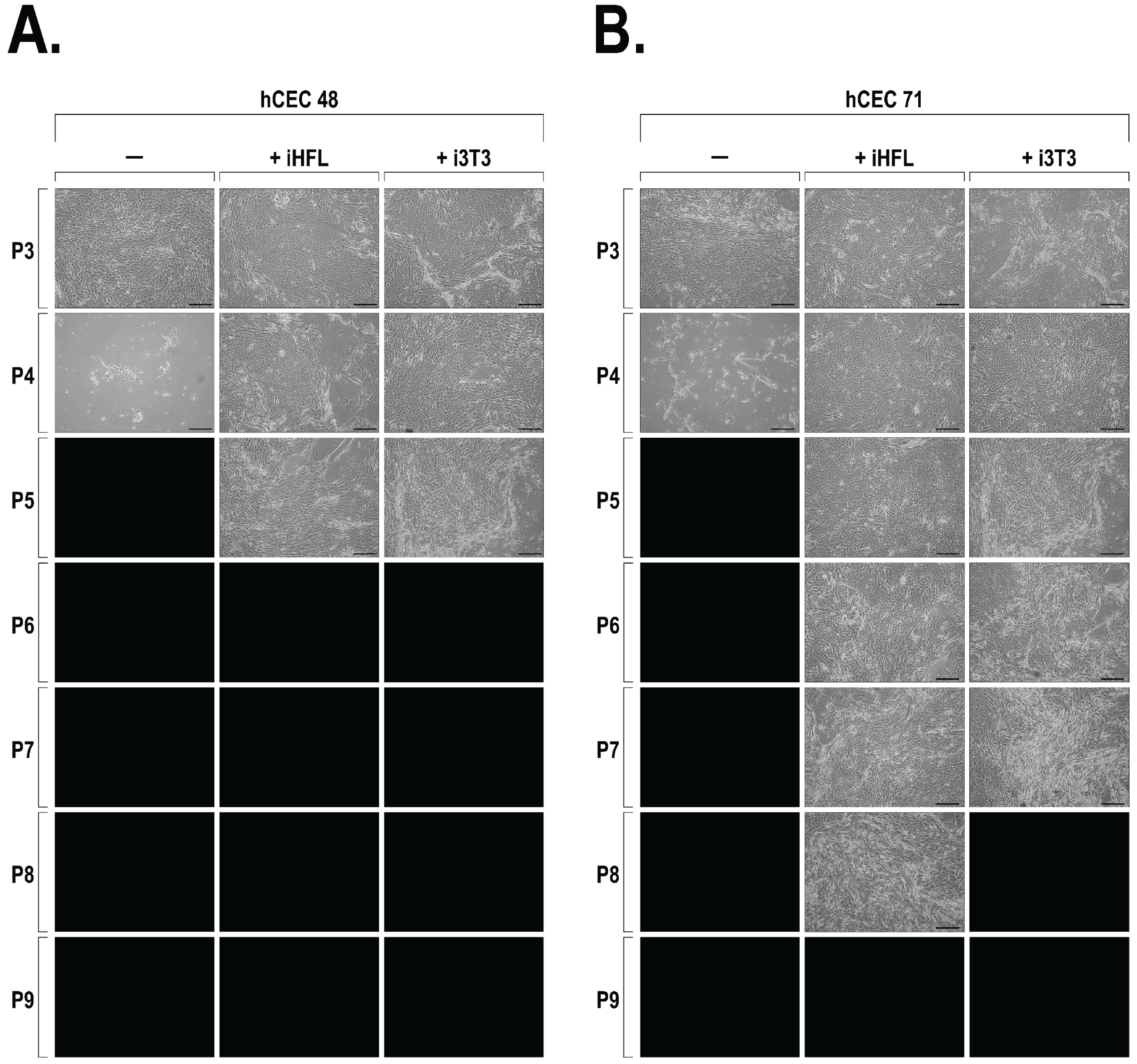

Supplementary Figure S1. Proliferative properties of hCECs grown with iHFL or i3T3 feeder layer. The human corneal epithelial cell populations hCEC48 (panel **A**) and hCEC71 (panel **B**) were grown either alone (-) or in the presence of a feeder layer (either iHFL or i3T3) until they reached near 90% confluence before they were trypsinized and re-plated. Cells were passaged until they reached replicative senescence.

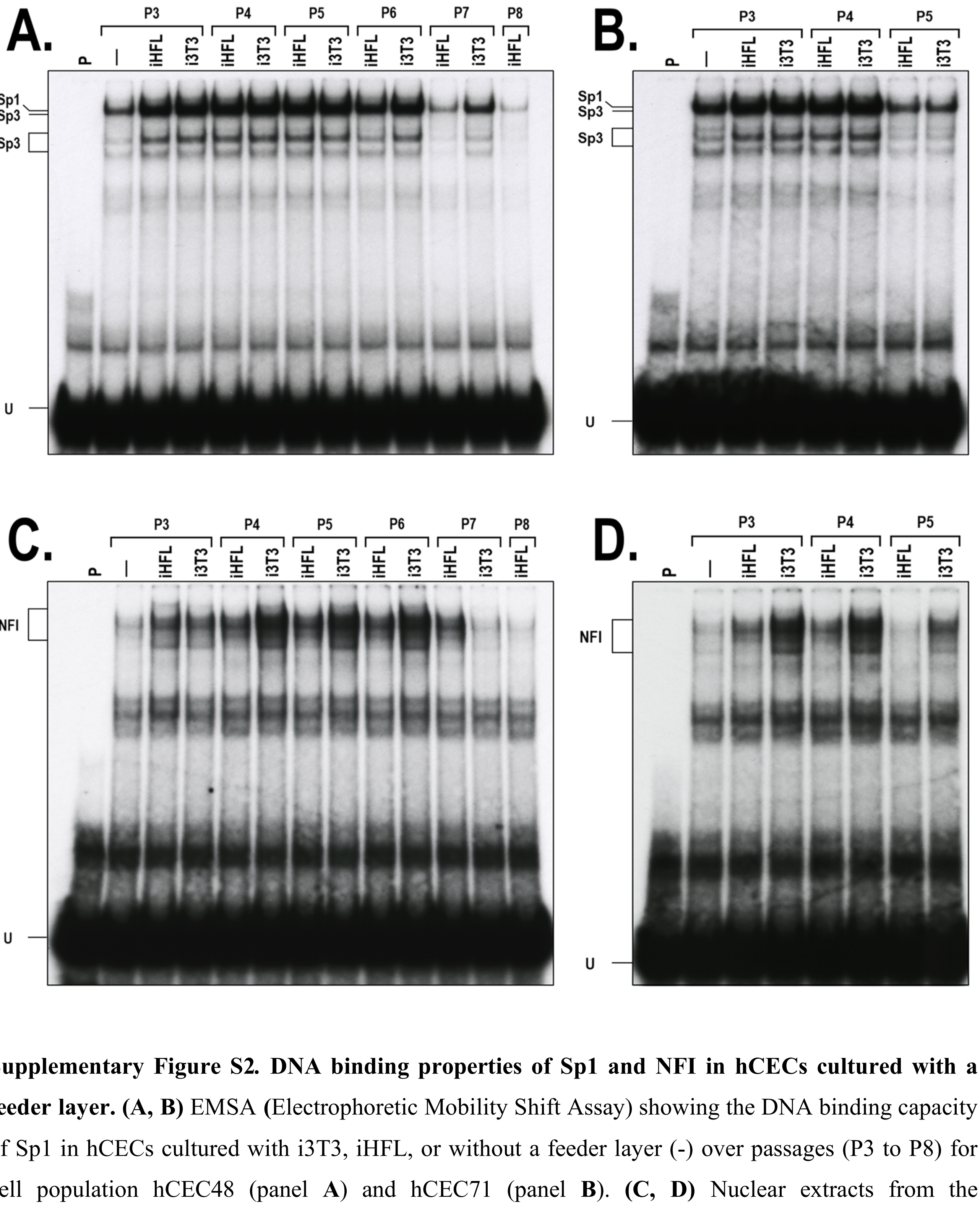

Supplementary Figure S2. DNA binding properties of Sp1 and NFI in hCECs cultured with a feeder layer. (A, B) EMSA **(**Electrophoretic Mobility Shift Assay) showing the DNA binding capacity of Sp1 in hCECs cultured with i3T3, iHFL, or without a feeder layer (-) over passages (P3 to P8) for cell population hCEC48 (panel **A**) and hCEC71 (panel **B**). **(C, D)** Nuclear extracts from the experiments of panel A and B were used to evaluate the capacity of NFI to bind to its high affinity target site in EMSA for cell population hCEC48 (panel **C**) and hCEC71 (panel **D**). P: labeled probe alone; U: unbound fraction of the labeled probe.

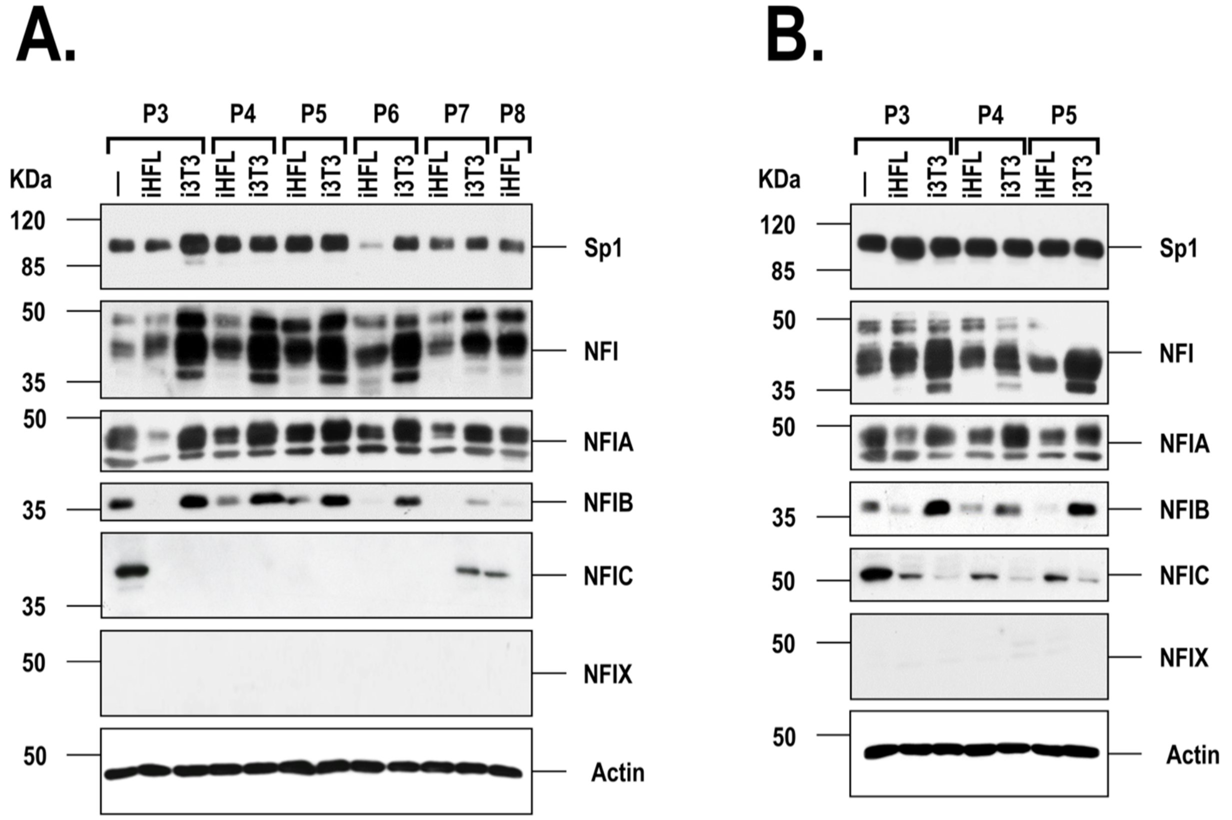

Supplementary Figure S3. Western blot analysis of Sp1 and NFI in hCECs cultured with a feeder layer. (**A**) Nuclear extracts prepared from hCEC48 (panel **A**) and hCEC71 (panel **B**) grown with or without (-) i3T3 or iHFL at different passages (P3 to P8) were separated by SDS-PAGE and Western blotted using antibodies raised against Sp1, each of the NFI isoform individually (NFIA, NFIB, NFIC and NFIX), or with an antibody that can recognize all isoforms (NFI). The position of the nearest molecular mass markers is indicated (KDa). Actin expression was also monitored as an internal control.

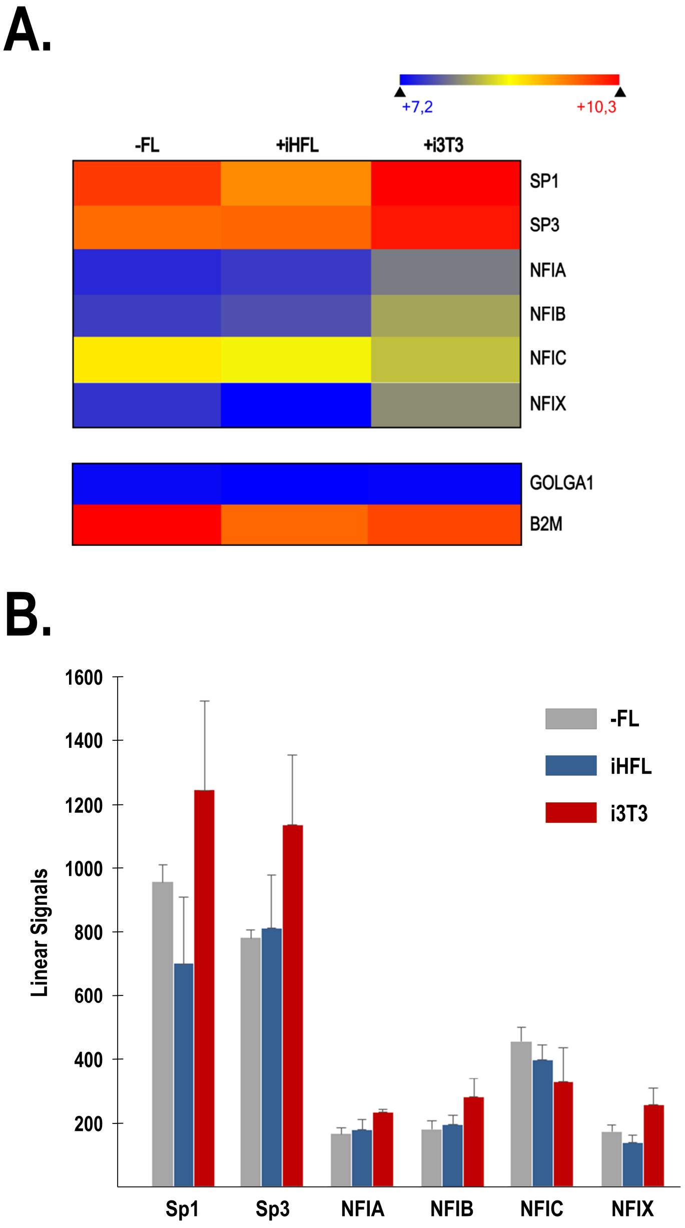

Supplementary Figure S4. Gene profiling analysis of the NFI isoforms expressed by hCECs cultured with a feeder layer. (**A**) Heatmap representation of the expression profiles of the genes encoding the transcription factors Sp1, Sp3 and all NFI isoforms between hCECs cultured with i3T3, iHFL, or without a feeder layer (-FL) at passage P3. Genes in dark blue are expressed to a very low level whereas highly expressed genes are in orange/red. (**B**) Graph representation of the linear signals corresponding to each of the genes analyzed in panel A.

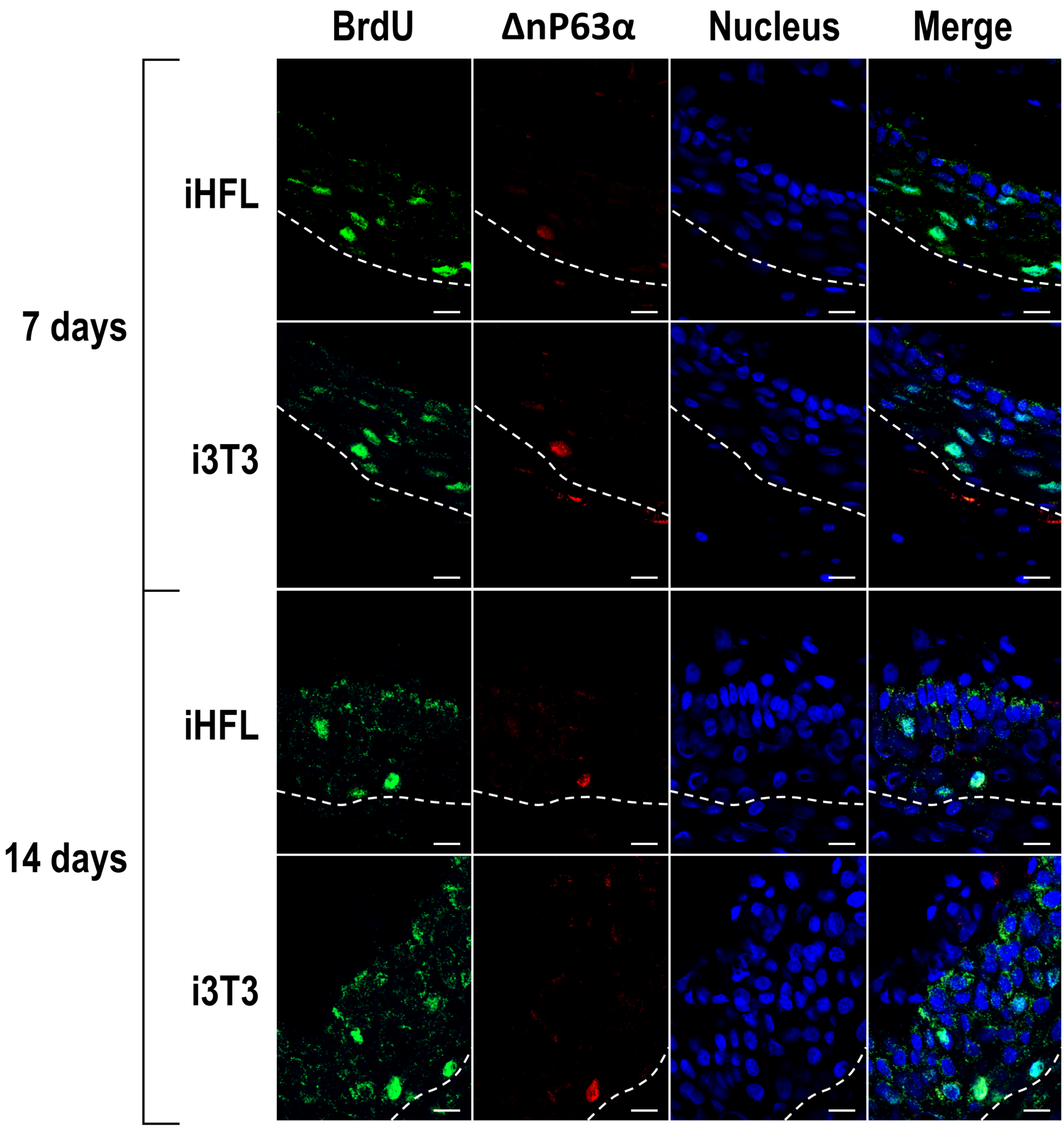

Supplementary Figure S5. Detection of human corneal epithelial stem cells in the tissue engineered cornea. Indirect immunofluorescence analysis of BrdU (green labeling) and ΔNp63α (red labeling) to assess the presence of stem cells in the basal layer of the tissue-engineered epithelia produced using hCECs grown with either i3T3 or iHFL as a feeder layer at 7 and 14 days following interruption of the BrdU treatment. Nuclei were counterstained with Hoechst 33258 reagent and appear in blue. The basal membrane is represented by a dotted line. Scale bar: 20µm.

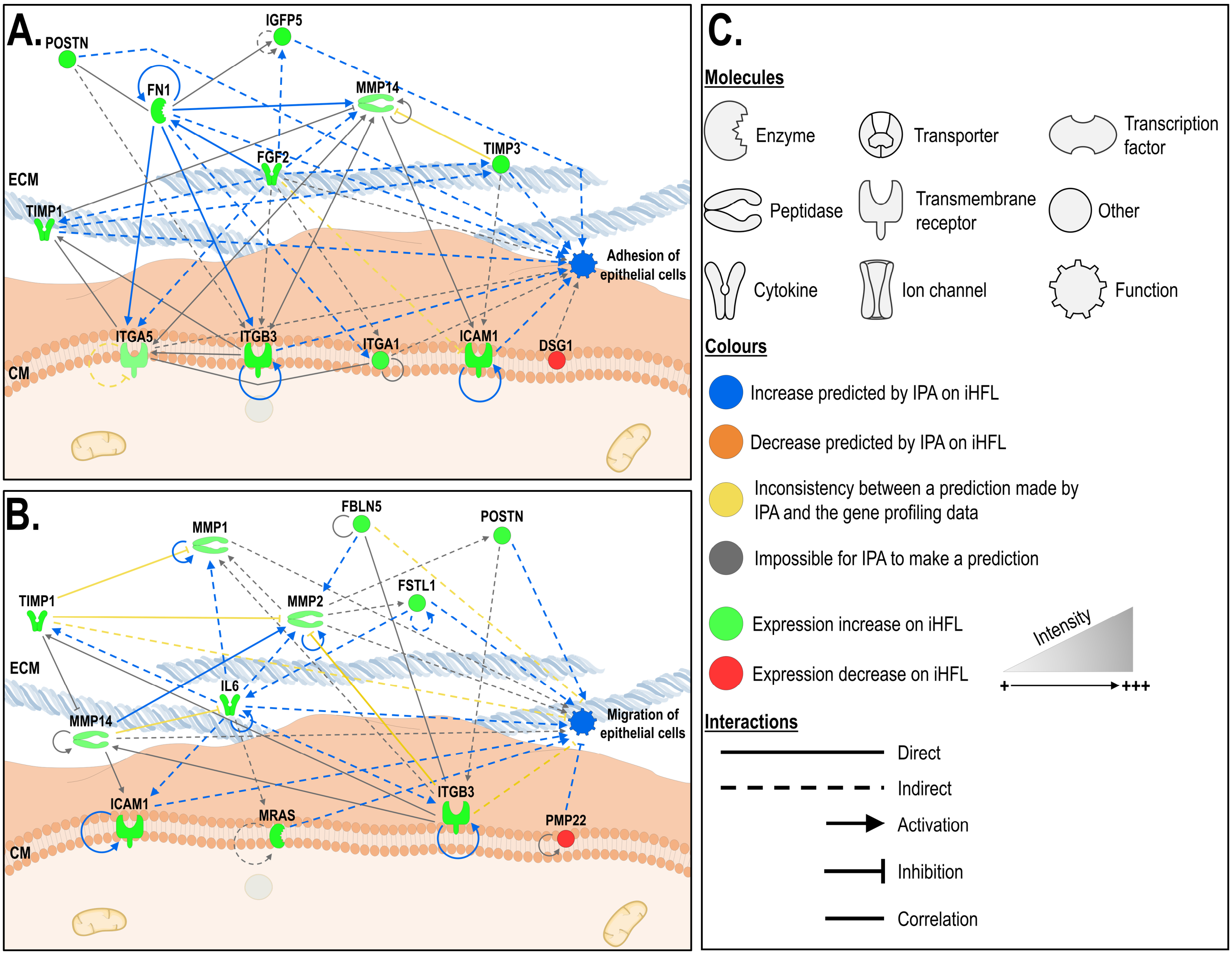

Supplementary Figure S6. Ingenuity pathway analysis (IPA) of gene interaction networks altered by the iHFL feeder layer built around biological functions of interest: the ‘adhesion’ (panel **A**) and ‘migration’ (panel **B**) of hCECs.
